# Functional assessment of the genetic findings indicating mucopolysaccharidosis type II in the prenatal setting

**DOI:** 10.1002/jmd2.12214

**Published:** 2021-03-26

**Authors:** Maria Fuller, David Ketteridge

**Affiliations:** ^1^ Genetics and Molecular Pathology, SA Pathology Women's and Children's Hospital North Adelaide South Australia Australia; ^2^ Adelaide Medical School University of Adelaide Adelaide South Australia Australia; ^3^ Metabolic Unit Women's and Children's Hospital North Adelaide South Australia Australia

**Keywords:** amniotic fluid, hunter syndrome, iduronate 2‐sulfatase, lysosomal storage disorder, mucopolysaccharidosis type II, prenatal diagnosis

## Abstract

Mucopolysaccharidosis type II (MPS II) is a multi‐systemic disorder arising due to pathogenic variants in the gene located on chromosome Xq28 encoding the lysosomal enzyme, iduronate 2‐sulfatase (IDS). The broad clinical heterogeneity of MPS II can be partly ascribed to the high level of molecular diversity in the gene locus with the majority of variants localised within one family. Here, we describe a case of fetal hepatomegaly that was causatively investigated for 151 genes associated with fetal hydrops and lysosomal diseases. Sequence analysis identified a novel hemizygous variant, pAsp532Gly, in exon 9 of the *IDS* gene. Determination of IDS activity in cultured amniotic fluid cells returned 8% of normal activity and analysis of a second sulfatase was normal, the latter virtually excluding multiple sulfatase deficiency. Together, these data supported a diagnosis of MPS II in the fetus. Additional measurement of a signature disaccharide in the amniotic fluid was normal, conflicting with enzymology indications. The baby was unremarkable at birth and 3 years later shows no clinical suspicion of MPS II, normal urinary disaccharide concentrations, and reduced IDS activity in leukocytes. His 5‐year‐old brother was subsequently shown to carry the same pAsp532Gly variant, with normal urinary disaccharide concentrations, reduced leukocyte IDS activity and normal phenotype. This case highlights the importance of thorough biochemical investigations, clinical and family correlation in determining the significance of genetic variants in *IDS*.

**Synopsis**

In a case of fetal hepatomegaly orthogonal validation of a novel hemizygous variant of uncertain significance, initially predicted to be likely pathogenic with reduced enzyme activity, was revised based on the absence of the enzyme's substrate, which supported the normal phenotype observed in the infant.

## 1 INTRODUCTION

Hunter syndrome or mucopolysaccharidosis type II (MPS II) is an X‐linked lysosomal storage disorder defined by a deficiency of iduronate 2‐sulfatase (IDS) responsible for the proper catabolism of the two glycosaminoglycans (GAG), dermatan sulfate and heparan sulfate. Accordingly, these partially degraded substrates accumulate in the lysosomes of affected cells, are associated with a chronic multisystemic disease and are excreted in the urine.^1^ Clinical manifestations typically include coarse facial features, skeletal and joint deformities, cardiopulmonary and respiratory insufficiency with intellectual disability in two thirds of patients. The signs and symptoms of MPS II typically appear from 2 to 4 years of age and for the majority of patients, delayed development is often apparent by the second year of life with progressive cognitive decline from 4 to 6 years of age.^2^

Following these clinical indicators, a diagnosis of MPS II is ordinarily achieved from laboratory investigations that return elevated GAG in the urine and reduced IDS activity in cells; leukocytes, dried blood spots or cultured skin fibroblasts are most commonly employed. Elevated GAG can be detected by simple dye‐binding assays,^3^ although more recently the use of mass spectrometry platforms has improved both the sensitivity and specificity of GAG detection. This typically occurs through chemical or enzymatic depolymerization of the high molecular weight GAG to disaccharides for mass spectrometric detection.^4^, ^5^ Laboratory determinations of IDS activity can be performed using a radiolabeled substrate consisting of a sulfated iduoronic acid linked glycosidically to a tritium labeled sulfated anyhdromannitol. Following IDS activity, the radioactive products are separated (chromatographically or electrophoretically) and quantified by scintillation counting.^6^ The use of a fluorimetric assay for IDS activity has simplified the laboratory measurement with the artificial substrate, 4‐methylumbelliferyl *α*‐L‐iduronide 2‐sulfate. This is a two‐step reaction that requires the addition of *α*‐L‐iduronidase in the second step after IDS has removed the sulfate residue in the first step, yielding the fluorescent product, 4‐methylumbelliferyl.^7^ Mass spectrometric detection of the products of IDS activity is also now possible and this technology has underpinned newborn screening programs.^8^ The protocol involves a synthetic IDS substrate and the products detected and quantified by reference to an internal standard using electrospray ionisation‐tandem mass spectrometry.^9^ Improved substrate design has led to increased sensitivity by generating a single product for detection following IDS activity.^10^

Although most MPS II patients will have complete absence or very little detectable IDS, some patients with a more attenuated phenotype can have up to 15% residual enzyme activity.^11^ Furthermore, the presence of pseudodeficiency alleles, which appear more common than true IDS deficiency, necessitates molecular testing to identify the pathogenic, disease causing variant before attributing a diagnosis of MPS II.^8^ Over recent years, the implementation of next generation sequencing (NGS) has afforded a window into MPS II diagnosis in an untargeted manner. Post‐hoc filtering allows for the creation of a virtual gene panel tailored to the clinical picture. Here, we describe the importance of orthogonal validation of genomic findings in a case of fetal hepatomegaly.

## 2 METHODS

### 2.1 Genetic testing

DNA was prepared from amniotic fluid collected at 31 weeks gestation following hepatomegaly on ultrasound (59 mm). NGS was performed using the Roche NimbleGen SeCap EZ MedExome capture on the Illumina NextSeq Sequencing System. Lysosomal storage disorder genes (71 of the total 151 genes analyzed previously associated with hepatomegaly and fetal hydrops)^12^, ^13^ included *AGA*, *ANTXR2*, *ARSA*, *ARSB*, *ASAH1*, *ASPA*, *ATP13A2*, *CLN3*, *CLN5*, *CLN6*, *CLN8*, *CTNS*, *CTSF*, *CTSA*, *CTSD*, *CTSK*, *DNAJC5*, *FUCA1*, *GAA*, *GALC*, *GALNS*, *GAMT*, *GBA*, *GCDH*, *GLA*, *GLB1*, *GM2A*, *GNE*, *GNPTAB*, *GNPTG*, *GNS*, *GRN*, *GUSB*, *HEXA*, *HEXB*, *HGSNAT*, *HYAL1*, *IDS*, *IDUA*, *KCTD7*, *LAMP2*, *LIPA*, *MAN2B1*, *MCOLN1*, *MFSD8*, *NAGA*, *NAGLU*, *NEU1*, *NPC1*, *NPC2*, *PEX1*, *PEX3*, *PEX5*, *PEX6*, *PEX10*, *PEX12*, *PEX13*, *PEX14*, *PEX16*, *PEX19*, *PEX26*, *PHYH*, *PPT1*, *PSAP*, *SGSH*, *SLC17A5*, *SMPD1*, *SUMF1*, *TPP1* and *VPS33A*. Variants were curated using Variant Studio v3.0, and those with a minor allele frequency > 0.1% in the population databases were excluded.

### 2.2 Enzyme activity and substrate determination

Cultured amniocytes were established from amniotic fluid and harvested cell pellets were sonicated in 0.1% Triton X‐100. For IDS activity, 0.15 mg of total cell protein—determined by the method of Lowry et al^14^—was first incubated with 4‐methylumbelliferyl‐*α*‐L‐iduronide‐2‐sulfate (Moscerdam Substrates, Oegsteest, Netherlands) for 4 hours at 37°C, followed by a second 24 hour incubation at 37°C with a pool of lysosomal enzymes from bovine testis (LEBT‐M2, Moscerdam Substrates) as described previously.^7^ The only exception was that the reaction was terminated with the addition of 200 μL of glycine buffer (0.2 M glycine, 0.125 M Na_2_CO_3_; 0.16 M NaOH; pH 10.7). Fluorescence was determined on a Walac Victor2 1420 multilabel plate reader (PerkinElmer, Waltham, MA) with an excitation wavelength of 366 nm and an emission wavelength of 446 nm. IDS activity was calculated by relating the fluorescence of the sample to that of a known concentration of 4MU. *N*‐acetyl galactosamine 6‐sulfatase was measured using the protocol provided by the substrate vendor (Moscerdam Substrates; Protocol M4A, March 2013).^15^

The equivalent of 0.5 μmoles of creatinine in urine and 50 μL of amniotic fluid were lyophilised and combined with 100 μL of 1‐phenyl‐3‐methyl‐5‐pyrazolone (0.25 M in 0.4 M NH_4_OH; pH 9.5) with 200 pmol of ΔUA‐GalNAc‐4S as the internal standard. Samples were incubated for 90 minutes at 70°C prior to acidification with 0.5 mL of 0.2 M HCOOH. Excess 1‐phenyl‐3‐methyl‐5‐pyrazolone was removed with CHCl_3_, and the signature MPS II disaccharide quantified by LC‐ESI‐MS/MS as described previously.^16^

## 3 RESULTS AND DISCUSSION

### 3.1 Novel variant in *IDS*

Sequence analysis of 151 genes associated with fetal hydrops and lysosomal storage disorders produced an overall estimated sensitivity of 98% variant detection, identifying a VUS, pAsp532Gly in exon 9 of the IDS gene. The IDS gene consists of nine exons and more than 600 different types of pathogenic variants have been reported as causative for MPS II. The main variants include point, frameshift, insertion, splice site, and minor and complete deletion of the IDS gene. This novel single nucleotide variant involves an A > G nucleotide change at cDNA position 1595 (NM_00202.7:c1595A > G), which is predicted to result in the substitution of an aspartic acid with a glycine residue. There is a physiochemical difference between these two amino acids, giving a Grantham score of 94 (range 0‐215) with lower scores associating with conservative amino acid substitutions. This variant is absent from the Genome Aggregation Database (gnomAD) and is moderately conserved across species. Bioinformatic analysis using the SIFT (v6.2.0), Mutation Taster (2013), PolyPhen‐2 and CADD suggest that p.Asp532Gly is likely to be pathogenic. Despite Align GVGD (2007) predicting this p.Asp532Gly likely benign, given that variants in *IDS* have been shown to be associated with fetal hepatomegaly,^17^ a diagnosis of MPS II was probable and biochemical investigations were subsequently undertaken to determine the significance of this variant.

### 3.2 Biochemical, clinical and family assessments provide orthogonal validation

Table 1 shows that there was measurable, but significantly reduced, IDS activity (11 nmol/4 hours/mg) in cultured amniocytes compared to the mean activity (132 nmol/4 hours/mg) from five unaffected controls. As no MPS II cultured amniocytes was available for testing, cultured MPS II skin fibroblasts and chorionic villus were included along with unaffected controls for quality assurance purposes, both showing that the MPS II samples had reduced IDS activity (Table 1). Typically, MPS II patients have no detectable IDS activity,^18^ and newborn screening studies have used 5% to 10% of daily mean of normal,^8^, ^17^ therefore, based on the finding of 8% residual enzyme activity it was predicted that this either resulted in an attenuated phenotype, or no phenotype at all. Multiple sulfatase deficiency was ruled out with normal *N*‐acetyl galactosamine 6‐sulfatase activity. Further laboratory investigations measured a GAG‐derived disaccharide believed to contain a terminal 2‐sulfated iduronic acid residue (the substrate for IDS), which was undetectable in the amniotic fluid (Table 1). This finding strongly suggested that the p.Asp532Gly variant was indeed benign as this disaccharide has been shown to be elevated in MPS II and specific for type II only (Table 1).^17^ At birth, the male infant was phenotypically normal, had no detectable disaccharide in his urine, and reduced leukocyte IDS activity (Table 1). His older brother (5 years of age at the time of his brother's birth) also harbored the p.Asp532Gly variant, had no detectable disaccharide in his urine, reduced leukocyte enzyme activity (Table 1) and was phenotypically normal.

**TABLE 1:** Biochemical findings

Biochemical parameter	Reference interval	Index case	Older sibling^a^	MPS II
Amniotic fluid IDS activity (nmol/4 hours/mg protein)	90‐170	11.0	N/A	N/A
CVS IDS activity (nmol/4 hours/mg protein)	97‐177	N/A	N/A	0.2, 1.0
SF IDS activity (nmol/4 hours/mg protein)	18‐69	N/A	N/A	0.5
Leukocyte IDS activity (nmol/4 hours/mg protein)	23‐80	6	5.5	N/A
*N*‐acetylgalactosamine 6‐sulfatase (nmol/17 hours/mg protein)	70‐265	256	155	N/A
Amniotic fluid disaccharide (nmol/mL)	<0.04	<0.04	<0.04	N/A
Urine disaccharide (mmol/mol creatinine)	<0.04	<0.04	<0.04	0.13‐0.31

Abbreviations: CVS, chorionic villus sampling; IDS, iduronate 2‐sulfatase; N/A, not available; SF, cultured skin fibroblasts.

^a^ Older sibling of index case aged 5 years at the time of testing.

Skeletal surveys in both siblings were normal. Mother was confirmed as a carrier of the p.Asp532Gly variant. There was a negative family history for any paternal relatives with a phenotype suggestive of MPS II. Both the proband and his elder brother have been monitored clinically and biochemically with no symptoms or signs of MPS II developing in the 3 years since the birth of the proband. Both siblings have macrocrania, which is believed to be benign familial macrocrania based on a positive family history of macrocrania in both parents', normal skull X‐ray and benign cranial ultrasound investigations.

## 4 CONCLUSION

This report highlights the importance of orthogonal validation of VUS in MPS II. Genetic investigations into a case of fetal hepatomegaly identified a VUS in IDS predicted to be likely pathogenic based on bioinformatic analysis alone. Reduced IDS activity (8% of the mean of normal) indicated a diagnosis of attenuated MPS II was indeed plausible, however the measurement of the enzyme's substrate was within the reference interval supporting the normal phenotype observed in the infant. The importance of genetic counseling for this family is underscored by the need to confirm or exclude MPS II and the associated anxiety this causes.

## CONFLICT OF INTEREST

Maria Fuller and David Ketteridge have no conflicts of interest to declare.

## AUTHOR CONTRIBUTIONS

Maria Fuller gathered the biochemical data and David Ketteridge gathered the clinical data on this family. Maria Fuller wrote the manuscript and both authors critically reviewed the final version.

## ETHICS APPROVAL

The use of patient samples in this study was approved by the Women's and Children's Health Network Human Research Ethics Committee (HREC/15/WCHN/69).

## INFORMED CONSENT

All procedures followed were in accordance with the ethical standards of the responsible committee on human experimentation (institutional and national) and with the Helsinki Declaration of 1975, as revised in 2000. All procedures were in accordance with routine patient care and the family consented to the publication of this report.

## ANIMAL ETHICS APPROVAL

This article does not contain any studies with animal subjects performed by any of the authors.
